# Response of *Chloris truncata* to moisture stress, elevated carbon dioxide and herbicide application

**DOI:** 10.1038/s41598-019-47237-x

**Published:** 2019-07-24

**Authors:** S. L. Weller, S. K. Florentine, N. K. Mutti, Prashant Jha, Bhagirath S. Chauhan

**Affiliations:** 1 Centre for Environmental Management, School of Health and Life Sciences, Federation University Australia, Mt Helen, Ballarat, PO Box 663, Vic, 3350 Australia; 2 Centre for Crop Science, Queensland Alliance for Agriculture and Food Innovation (QAAFI), The University of Queensland, Gatton, Queensland 4343 Australia; 3 Department of Agronomy, Iowa State University, Ames, IA 50011 United States of America

**Keywords:** Plant sciences, Plant development

## Abstract

Herbicide resistance has been observed in *Chloris truncata*, an Australian native C_4_ grass and a summer-fallow weed, which is common in no-till agriculture situations where herbicides are involved in crop management. To investigate the role of drought and increased atmospheric carbon dioxide (CO_2_) in determining weed growth, three trials were conducted using a ‘glyphosate-resistant’ and a ‘glyphosate-susceptible’ biotype. The first two trials tested the effect of herbicide (glyphosate) application on plant survival and growth under moisture stress and elevated CO_2_ respectively. A third trial investigated the effect on plant growth and reproduction under conditions of moisture stress and elevated CO_2_ in the absence of herbicide. In the first trial, water was withheld from half of the plants prior to application of glyphosate to all plants, and in the second trial plants were grown in either ambient (450 ppm) or elevated CO_2_ levels (750 ppm) prior to, and following, herbicide application. In both biotypes, herbicide effectiveness was reduced when plants were subjected to moisture stress or if grown in elevated CO_2_. Plant productivity, as measured by dry biomass per plant, was reduced with moisture stress, but increased with elevated CO_2_. In the third trial, growth rate, biomass and seed production were higher in the susceptible biotype compared to the resistant biotype. This suggests that a superior ability to resist herbicides may come at a cost to overall plant fitness. The results indicate that control of this weed may become difficult in the future as climatic conditions change.

## Introduction

*Chloris truncata* R.Br. is an Australian native C_4_ (warm season) short-lived perennial grass, and it has recently become of significant concern in no-till agriculture conditions^1,2^. Originally thought to be native to Queensland, New South Wales and Victoria^3^, its range has expanded into the more southerly and western parts of the Australian continent, which is most likely due to year-round increases in average temperatures^4^.

This species is also naturalized in southern Africa, North America and New Zealand^5^; however, it is thought that climate change may impact the future distribution of this species^3^. Modelling changes to seasonal temperature ranges and rainfall have indicated a reduction in the area of suitable climate where it occurs as a native species^3^, which may lead to population reductions within these regions. In addition, as a potentially positive outcome for the regions in which this species is regarded as an exotic weed, the total area of suitable climate zone in other parts of the world may also be significantly reduced. Alternatively, it may spread to other localities, such as southern and south-western Europe and central China, as the climate in these regions becomes more suitable^3^. Notwithstanding these predictions, data on the biological effects of increased atmospheric CO_2_, drought and temperature changes for this species is currently lacking. Therefore, conclusions from the previously used modelling data for predicting changes in the range of occurrence of *C. truncata* should perhaps be treated with caution.

Seeds of this species are produced from spring to autumn^5^, and the resulting seed bank does not appear to be particularly long-lived. Maximum germination occurs following an after-ripening period of five to seven months^2^. Seeds have a maximum lifespan of one to two years in the soil^1,2^, although a slightly longer lifespan under dry conditions, extended by approximately six months, has been observed in some populations^6^. Optimal temperature range for germination is 20 °C to 25 °C, but seeds may germinate in temperatures as low as 10 °C or as high as 30 °C^2,6,7^. Light is important for germination, and low moisture availability both reduces germination percentage and delays its onset^2^. A lack of moisture is, therefore, more limiting on germination than temperature^6^. Since this plant does not generally live more than one season and the seeds are not very long-lived in the soil, it is likely that active intervention will reduce infestations to more manageable levels in one to two years.

However, it has been discovered that this species^2^ and others in the same genus^8–11^ are becoming resistant to glyphosate, a commonly used herbicide of cropping regions and other weed infested areas such as roadsides^12^. This is part of an overall trend exhibited by many species recognised as agricultural and environmental weeds, where the widespread use of this herbicide is driving not only the evolution of individual weed species but is also affecting the ecological relationships between weed species^13–16^. Investigations into why this process is occurring have included attempts to determine which specific resistance mechanisms are responsible for the observed changes in herbicide resistance, and this includes attention to genomic factors^2,8,10,17^.

It has been observed that adaptive responses of plants to environmental stresses such as a drought, appear to result in increased resistance of weeds to herbicides. The most likely mechanism responsible for this relationship is a change in leaf water conductance, where a reduction in soil moisture stimulates the closure of leaf stomata^18,19^. However, it is suspected that increased herbicide resistance under this circumstance is merely coincidental. Whether changes in leaf water conductance are caused solely by moisture stress or whether increased atmospheric CO_2_ also plays a role in this process, is an issue yet to be confirmed.

In this respect, it is known that increased atmospheric CO_2_ affects plant growth and metabolism, according to metabolic pathway type, which may be either C_3_ or C_4_. Whether the functional characteristics possession of one of these metabolic pathways is more likely than the other to lead to herbicide resistance with climate change, and what role increasing atmospheric CO_2_ plays in this process, is not yet completely understood^20^. However, it is clear that net assimilation of CO_2_ is relatively larger in C_3_ than in C_4_ grasses as atmospheric concentrations increase^18^. The outcome of higher concentrations of CO_2_ within the leaf tissues is an increase in metabolic rate, resulting in faster growth. Therefore, resistance to herbicides of C_3_ species grown at elevated CO_2_ may be due, at least in part, to increased biomass production^21^.

By contrast, C_4_ plants are not expected to gain a significant advantage, in terms of increase in metabolic response, to elevated CO_2_, since they should (theoretically) not be expected to increase the amount of CO_2_ within leaf tissues unless there is a corresponding increase in the amount of available oxygen^22^. Nonetheless, some C_4_ grass species have been found to significantly increase biomass when exposed to elevated CO_2_^23^, which is indicative of an increased metabolic response. This increase in overall plant size may lead to dilution of herbicide levels within plant tissues, in a similar manner to that which has been observed in C_3_ plants, and therefore this may reduce the efficacy of herbicides. Physiological changes to leaf tissues, such as a thickening of the leaf cuticle, have also been recorded in C_4_ plants exposed to elevated CO_2_ levels^24^, and these may also reduce the uptake of herbicide by C_4_ plants. However, the precise biochemical and genetic mechanisms triggered by increased atmospheric CO_2_ that relate to changes in herbicide resistance in C_4_ plants are not yet completely resolved. Therefore, it is unlikely that determining exactly which factors are responsible for this phenomenon will be a straightforward matter.

Additionally, it is important to emphasise that although herbicide resistance is sometimes discussed as though an entire species has become resistant to herbicides simultaneously, this is usually not the case. This trait is more likely to be confined to populations of weed species that possess a high degree of genetic diversity and possibly arises as a result of the use of herbicides at sub-lethal concentrations. Such conditions are likely to be the first step towards subsequently stronger, and more widespread, resistance within such populations^25^. Other research has attempted to uncover specific mechanisms responsible for herbicide resistance, including quantifying the degree of resistance according to population source^2,8,10,17^. However, a comparison of populations from within the same species that are known to be susceptible to herbicide with those that are known or suspected to be resistant does not appear to have been given as much attention. Such comparisons may allow for identification of whether or not recently evolved herbicide resistance, traits impact on plant fitness in a more general sense^26^. This may lead to other relevant understandings, including how to respond to herbicide-resistant weeds.

The aims of this research were to investigate the effect of (1) drought and (2) elevated CO_2_ on the effectiveness of glyphosate to control *C. truncata*, as well as (3) the effects of elevated CO_2_ and drought on plant growth, biomass, and seed production in the absence of glyphosate. Two biotypes, glyphosate-susceptible and resistant, were used to conduct this investigation. The results may be used to predict future changes in the distribution of this weed, as well as to inform future management of this weed in agricultural situations and in regions where environmental intervention is needed.

## Results

### Trial 1: Effect of water stress and herbicide

#### Survival and shoot dry matter

**Figure 1:**
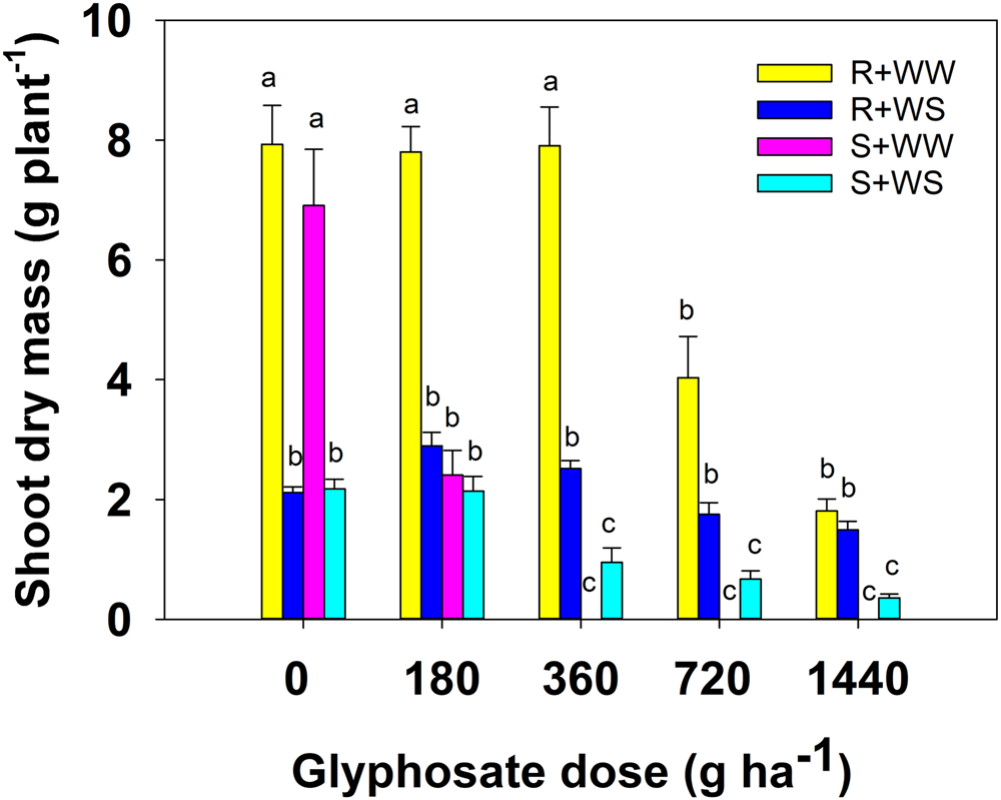
Effect of glyphosate dose (g a.e. ha^−1^) and moisture [well-watered (WW) or water stressed (WS) conditions] on shoot dry mass (g plant^−1^) of susceptible (S) and resistant (R) biotypes of *Chloris truncata*. Error bars are the standard error of means. Means with the same letter are not statistically different from each other.

All plants of the resistant biotype survived whether they were well-watered or not, and regardless of the herbicide application rate (Table S1). In the susceptible biotype, there was 100% survival of the well-watered (WW) plants in the control, 0 g a.e. ha^−1^ (g a.e. = grams of acid equivalent), and lowest herbicide application rate (180 g ha^−1^), but no other plants survived. However, in the water-stressed (WS) treatment, there was 100% survival of this biotype at all herbicide application rates. A three-way ANOVA indicated a significant difference between biotypes according to differences in either water stress or herbicide concentration (p < 0.001). Shoot dry matter of both biotypes was significantly lower in WS than in the WW treatment, and was also reduced with increasing herbicide application rate (Fig. 1).

The mean values for shoot dry matter according to each factor (biotype, water treatment or herbicide application rate) are given in Table 1. Overall, shoot dry matter was higher for the resistant biotype plants compared to the susceptible biotype, but water stress significantly reduced biomass for both biotypes. Biomass was highest with no herbicide treatment and decreased steadily with increasing herbicide application rate.

**Table 1:** Effect of biotype, water treatment [well-watered (WW) or water stressed (WS) conditions] and glyphosate dose (g a.e. ha^−1^) on shoot dry matter (g plant^−1^) of susceptible (S) and resistant (R) biotypes of *Chloris truncata*. Values in parentheses are the standard error (SE) of means.

Treatments		Shoot dry matter (g plant^−1^) (±SE)	p-value
Biotype	Resistant	4.02 (±0.32)	<0.001
	Susceptible	1.56 (±0.25)	
Water treatment	Water-stressed	1.71 (±0.10)	<0.001
	Well-watered	3.88 (±0.40)	
Glyphosate dose (g a.e. ha^−1^)	0	4.78 (±0.54)	<0.001
	180	3.81 (±0.44)	
	360	2.84 (±0.57)	
	720	1.61 (±0.32)	
	1440	0.91 (±0.15)	

### Trial 2: Effect of elevated carbon dioxide and herbicide application

#### Survival and shoot dry matter

**Figure 2:**
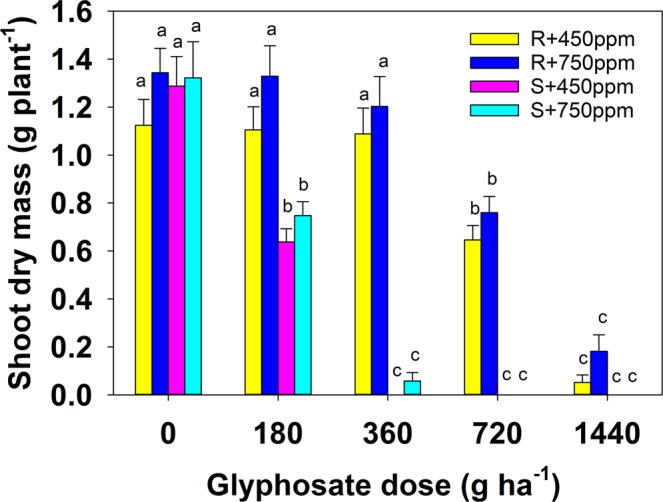
Effect of glyphosate dose (g a.e. ha^−1^) and carbon dioxide concentration (450 ppm and 750 ppm) on shoot dry mass (g plant^−1^) of susceptible (S) and resistant (R) biotypes of *Chloris truncata*. Error bars are the standard error of means. Means with the same letter are not statistically different from each other.

Increased CO_2_ significantly reduced the effectiveness of herbicide (p = 0.036), with both biotypes showing increased resistance to glyphosate in elevated (750 ppm) CO_2_ compared to ambient levels (450 ppm) (Table S2). Additionally, there was an increase in dry matter of plants of both biotypes in the presence of elevated CO_2_ compared to ambient conditions (Fig. 2).

A three-way ANOVA indicated significantly higher survival rates of the resistant biotype (<0.001). There was a statistically significant interaction between biotype, CO_2_ level and herbicide application (p < 0.001). In ambient CO_2_ conditions, all the susceptible plants in the control and lowest herbicide application rate survived, but the remainder did not. However, at elevated CO_2_, 33% of these plants survived the second lowest herbicide application rate (360 g ha^−1^). Survival of the resistant biotype was also increased in elevated CO_2_ conditions. In ambient CO_2_, 25% of plants survived the maximum application rate (1440 g ha^−1^). However, survival increased to 63% at the elevated CO_2_ level. It is noteworthy that this amount of herbicide is double the current recommended application rate.

Shoot dry matter was significantly greater for both biotypes according to herbicide application rate (p < 0.001) and exposure to elevated CO_2_ (p = 0.011). At the highest herbicide application rate, biomass of resistant plants was significantly greater at elevated CO_2_ than in ambient. There was a statistically significant interaction between biotype and herbicide application rate, but not between CO_2_ and biotype, nor between CO_2_ and herbicide application rate.

The mean values for shoot dry matter according to each factor were lower at elevated CO_2_ and herbicide conditions (Table 2) compared with water stress and herbicide application (Table 1). The resistant plants produced twice as much biomass as the susceptible biotypes, and elevated CO_2_ also resulted in significantly more biomass production overall. However, as was found in the water and CO_2_ trial, increasing amounts of herbicide reduced shoot dry matter.

**Table 2:** Effect of biotype, carbon dioxide (450 and 750 ppm) and and glyphosate dose (g a.e. ha^−1^) on shoot dry matter (g plant^−1^) of susceptible (S) and resistant (R) biotypes of *Chloris truncata*. Values in parentheses are the standard error (SE) of means.

Treatments		Shoot dry matter (g plant^-1^) (±SE)	p-value
Biotype	Resistant	0.88 (±0.06)	<0.001
	Susceptible	0.41 (±0.06)	
CO_2_ level	450 ppm	0.59 (±0.06)	0.011
	750 ppm	0.69 (±0.07)	
Glyphosate dose (g a.e. ha^−1^)	0	1.27 (±0.06)	<0.001
	180	0.95 (±0.07)	
	360	0.59 (±0.11)	
	720	0.35 (±0.07)	
	1440	0.06 (±0.02)	

### Trial 3: Effect of carbon dioxide and water stress

#### Plant height

There was faster initial growth in the resistant biotype after the commencement drought treatment, since the resistant plants were slightly taller than susceptible varieties in Weeks 0 and 2 (Table S3). However, this difference was largely overcome by Week 5, with less difference in height between the plants of each biotype. At the conclusion of the experiment in Week 7, the susceptible plants were taller than the resistant plants in all treatments (Susceptible biotype: 50% water, ambient = 66.4 ± 3.2 cm, elevated = 73.0 ± 5.4 cm; 100% water, ambient = 74.1 ± 2.3 cm, elevated = 74.5 ± 2.2 cm; and Resistant biotypes: 50% water, ambient = 58.2 ± 2.1 cm, elevated = 64.2 ± 3.5 cm; 100% water, ambient = 57.7 ± 1.2 cm, elevated = 68.4 ± 3.5 cm). Although a lack of moisture did not significantly reduce the overall growth rate in either, it was slightly less in the 50% water treatment compared to the 100% condition.

### Number of leaves

At Week 0, there was little difference in the number of leaves produced by all plants (Table S4). However, the resistant plants had more leaves than the susceptible varieties in most treatments, although the differences were not statistically significant (p > 0.001). The profile was similar in Week 2, with the resistant biotype plants producing more leaves in the 100% water and elevated CO_2_ treatments than the susceptible plants. By Week 5, there was a greater divergence between each biotype, but it was found that the susceptible plants had now produced more leaves than the resistant biotype. At Week 7, the susceptible plants (Susceptible biotypes 50% water, ambient = 136.5 ± 7.7, elevated = 140.7 ± 9.6, 100% water, ambient = 164.5 ± 14.2, elevated = 167.0 ± 17.2) had clearly produced more leaves than the resistant biotypes (Resistant biotype: 50% water, ambient = 83.7 ± 5.8, elevated = 93.5 ± 4.8, 100% water, ambient = 117.7 ± 10.5, elevated = 132.2 ± 17.7) and this difference was statistically significant (p < 0.001).

### Number of inflorescences

None of the plants had inflorescences at Week 0 (Table S5). The susceptible plants were subsequently slower to commence inflorescence development than the resistant group. By Week 2, the resistant plants had produced a total of four inflorescences, two in each of the water treatments in elevated CO_2_. The susceptible plants developed inflorescences by Week 5, but the resistant plants had produced more inflorescences in the 50% water and ambient CO_2_ treatment, and in both water treatments in elevated CO_2_. By Week 7, the susceptible plants had produced more inflorescences than the resistant biotype in ambient CO_2_ and both water treatments (Susceptible biotype: 50% water, ambient = 6.5 ± 0.7, elevated = 6.2 ± 1.1, 100% water, ambient = 7.5 ± 1.9, elevated = 5.7 ± 1.6). Conversely, the resistant biotype plants had produced more inflorescences at elevated CO_2_ and both water treatments (Resistant biotype: 50% water, ambient = 5.0 ± 0.8, elevated = 7.0 ± 0.5, 100% water, ambient = 5.2 ± 0.8, elevated = 5.8 ± 1.0). However, the total number of inflorescences at Week 7 was higher overall for the susceptible biotype plants (a total of 155 for all plants) than the resistant (a total of 138 for all plants).

A repeated measures ANOVA indicated no significant effect on plant height due to elevated versus ambient CO_2_, nor was there any significant effect on the number of inflorescences produced at each sampling time. There was also no significant difference in plant height with increased moisture stress between biotypes. However, the number of leaves did vary significantly according to biotype (p = 0.002) and water stress level (p = 0.03), with the resistant plants producing significantly fewer leaves than the susceptible plants, with water stress being the significant factor in this result.

### Shoot dry matter

**Figure 3:**
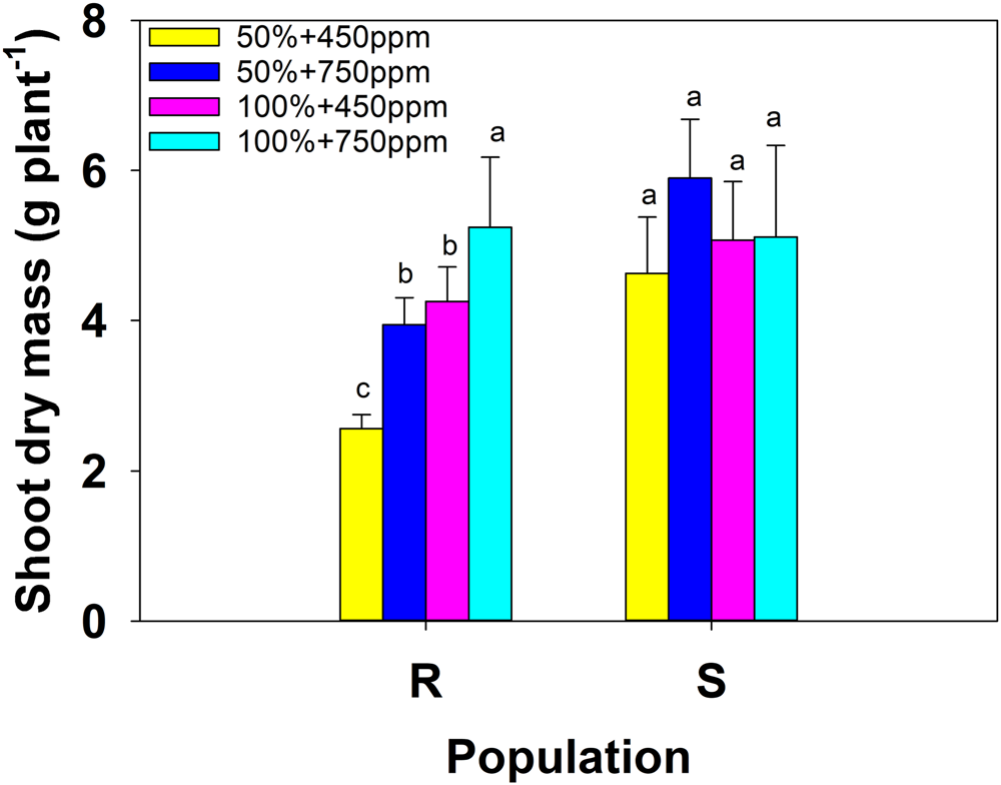
Effect of water regime (50% and 100% field capacity) and carbon dioxide on shoot dry mass (g plant^−1^) of susceptible (S) and resistant (R) biotypes of *Chloris truncata*. Error bars are the standard error of means. Means with the same letter are not statistically different from each other.

Shoot dry matter of the susceptible biotype plants was significantly higher in all water and CO_2_ treatments than the resistant biotype, except for the 100% water and elevated CO_2_ treatments (Fig. 3). The overall amount of shoot dry matter produced by the susceptible biotype was significantly higher than the resistant biotype.

### Inflorescence dry matter

The trends for inflorescence dry matter due to water stress and exposure to CO_2_ varied according to biotype (Table S6). Inflorescence dry matter was highest in the susceptible plants in 100% water and ambient CO_2_ (1.5 ± 0.3 g), and lowest in the resistant biotype in 50% water and the same CO_2_ treatment (0.8 ± 0.1 g). However, increased availability of CO_2_ increased inflorescence dry matter production in resistant plants but reduced inflorescence the same in susceptible plants. In resistant plants, the amount of inflorescence dry matter increased in elevated CO_2_, regardless of the amount of available moisture (change from ambient to elevated CO_2_: 50% water = 0.8 ± 0.1 g to 0.9 ± 0.1 g; 100% water = 0.9 + 0.2 g to 1.3 + 0.2 g). However, the opposite trend was observed in susceptible plants (50% water = 1.1 + 0.1 g to 0.8 + 0.2 g; 100% water = 1.5 + 0.3 g to 1.0 + 0.3 g). However, there was no significant difference in the amount of inflorescence dry matter produced by either biotype.

### Number of seeds

**Figure 4:**
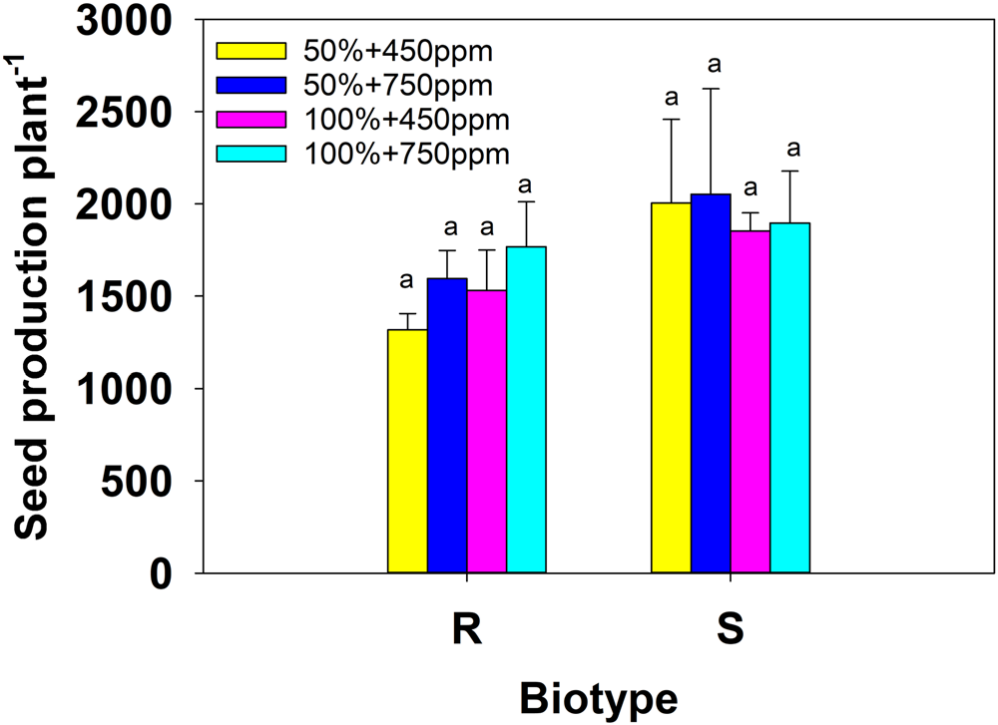
Effect of water regime (50% and 100% field capacity) and carbon dioxide on seed production (number plant^−1^) of susceptible (S) and resistant (R) biotypes of *Chloris truncata*. Error bars are the standard error of means. Means with the same letter are not statistically different from each other.

Seed production was higher overall in the susceptible biotype than the resistant biotype, but this difference was not statistically significant. The number of seeds produced by both biotypes was slightly higher in elevated CO_2_ conditions than ambient, but the effect of water contrasted slightly for each (Fig. 4). The resistant plants produced slightly more seeds per plant in 100% water than in the 50% condition in each CO_2_ treatment, but the susceptible plants produced slightly more seeds overall under water-stressed conditions, compared to no moisture stress.

## Discussion

### Trials 1 and 2: Effects of moisture stress and carbon dioxide on the efficacy of herbicide

The first recorded occurrence of glyphosate resistance of a weedy species, *Lolium rigidum* Gaud., was reported in Australia in 1996^27^. Since this time, many more weed species have begun to show resistance to this herbicide^15–17,28^, including some members of the genus *Chloris*^2,8–11^. Therefore, understanding how herbicide resistance of agricultural weeds occurs is perhaps one of the most important topics of research for agriculture today. Additionally, testing individual species of plants for their potential reactions to environmental stressors that are linked to climate change is also an active area of research, since there are some indications that drought and increasing concentrations of atmospheric CO_2_ are also changing plant responses to herbicides^23,29^. Linking observations of increased resistance to herbicides, due solely to exposure to herbicides, together with physiological changes that occur in response to climate factors but which may also alter responses to herbicide exposure, may provide evidence that these mechanisms of herbicide resistance are interrelated. Discovering whether this is the case may enable novel management strategies to be developed to address the current outcomes, or possibly suggest areas of research for future investigations into this problem to find alternate solutions to continued reliance on herbicides.

Some populations of the species investigated in this trial of *Chloris truncata* have been known to be herbicide resistant since 2010^30^. However, in the current study, this species demonstrated that even the herbicide-susceptible biotype of this weed appears to show some tendency towards reduced herbicide efficacy, given an appropriate stimulus. Increased survival to herbicide exposure under water stress and increased amounts of atmospheric CO_2_ were observed in the susceptible biotype, although the second of these observations was significantly lower than the first. In the water and herbicide experiment, 100% of the susceptible plants survived treatment with all herbicide concentrations when water stressed, compared to none surviving in the absence of moisture stress when treated with herbicide concentrations above the lowest amount of 180 g ha^−1^. In the CO_2_ and herbicide experiment, a comparatively modest increase in survival was observed in the susceptible biotype plants at elevated CO_2_. Exposure to herbicide application rates of 360 g ha^−1^ and higher were fatal in this biotype in the ambient CO_2_ treatment. However, two individuals (out of eight in total) survived this herbicide application rate at elevated CO_2_. This suggests that elevated CO_2_ has a benefit for the survival of this species, even if only a relatively small one. Other researchers have concluded that this effect is possible, but do not yet appear to have proffered an explanation for this resistance^23,29^. This indicates that further research should be undertaken to determine the precise mechanism of how elevated CO_2_ leads to this result.

In addition to survival following exposure to herbicide, measures of plant productivity in response to environmental stressors may be used to predict likely responses of weeds to climate change. For example, in the water and herbicide experiment, plant dry matter was significantly reduced in both biotypes in moisture-stressed conditions. This is perhaps not entirely unexpected, since moisture stress places limits on plant productivity, leading to reduced plant growth^31–33^. In the CO_2_ and herbicide experiment, by contrast, there was an increase in plant dry matter with elevated CO_2_.

To explain these observations, it is most likely that the increase in survival under water stress is due to the closure of leaf stomata in response to a lack of moisture. This has been observed previously and is an evolved trait of plants in response to hot, dry conditions^34^, which coincidentally reduce the possibility of herbicide uptake by the leaves. Additional factors that may explain the observed results for increased dry matter may include the phenological response of plants to elevated CO_2_ exposure^29,35–38^. For example, changes in leaf morphology during early growth, whereby leaf tissues become thickened, or through variation in the root to shoot ratio, may lead to an overall increase in plant size and weight. Also, elevated CO_2_ is known to reduce nitrogen and protein levels in the leaves, resulting in a reduction of the efficacy of glyphosate^39^. In addition to these changes, the increased size of the plants due to elevated CO_2_ may have had the effect of diluting herbicide concentration within the plant, thereby aiding plants’ survival to herbicide exposure^23^.

The mechanisms for resistance to glyphosate that have been found in this species^2^ and others in the same genus^8–11^ do not appear to function in exactly the same manner, are somewhat complex, and may contrast significantly within a species or change over time. The two possibilities for resistance to herbicides that have been identified are target site resistance (TSR) or non-target site resistance (NTSR) mechanisms. The TSR mechanisms appear to operate as a result of alteration of the DNA, either by amplification of the EPSPS (5-enolpyruvylshikimate-3-phosphate synthase) gene or point mutation, whereby gene substitution occurs. The first of these, amplification of the EPSPS gene, occurs when there is an increase in the number of copies of this gene within the DNA. This correlates with increased resistance to herbicides, and thus the specific increase in the number of gene copies is thought to determine the degree of resistance^2,11^. Substitution of particular genes alters the ability of the plant to resist herbicides, with either the type or number of these substitutions conferring differing degrees of resistance^10^. The NTSR mechanisms appear to operate due to physiological responses of plants when exposed to herbicides. These can be: (i) increasing plant size; (ii) alteration of leaf tissue to resist uptake of herbicide; (iii) action within the leaves which allows them to retain herbicide that is absorbed but in a manner that does not affect the plant overall; or (iv) translocation of herbicide to parts of the plant where it can do less damage^8,9^.

Previously studied resistant populations of the species used in this study (*C. truncata*) were found to use a TSR mechanism, namely amplification of the EPSPS gene^2^. This resistance mechanism has also been found in other weed species, for example, *L. rigidum*^17^. Another species in the genus *Chloris*, *C. virgata*, was also found to have also used a TSR mechanism. However, instead of gene amplification of the EPSPS gene, resistant populations of *C. virgata* had undergone gene-substitution mutations^10^. Of specific and crucial interest, is that the gene substitution was not the same in each of the four populations investigated by these researchers. In one population, there was a change in Pro-106-Leu, but in the remaining three populations, the change was for Pro-106-Ser. The outcome of these contrasting changes was to confer a higher level of resistance to the first of these populations compared to the remaining three. By contrast to the TSR mechanisms employed by *L. rigidum, C. virgata* and *C. truncata*, the herbicide-resistant populations of *C. elata* from Cuba^9^ and Brazil^8^ were found to use NTSR mechanisms for their herbicide resistance rather than amplification of EPSPS gene. This implies that although plants in the same genus may share some genetic characteristics in common, including how they respond to novel stressors such as exposure to herbicides, the finer details of these processes can be somewhat complicated. However, it is likely that the mechanism for resistance in the *C. truncata* plants in this study is an amplification of the ESPS gene. To confirm this conclusion, it is recommended that genetic analysis is carried out on any remaining seeds from this experiment, or that plants from the source populations be collected for such analysis.

### Trial 3: Effects of moisture stress and carbon dioxide on plant and inflorescence dry matter, and seed production

The effects of moisture stress and elevated CO_2_ in the absence of herbicide application highlighted some interesting differences between each of the two biotypes for their potential responses to climate change. However, there were no statistically significant differences between each, apart from for the number of leaves produced. This was significantly higher in the susceptible biotype than the resistant group, and the most likely cause for this difference was due to the watering regime, although elevated CO_2_ may have also influenced this result^40^. Otherwise, in terms of plant productivity, flowering effort and seed production, differential water stress or CO_2_ concentration did not significantly advantage either biotype, even if it did initially appear to be the case.

Notwithstanding this observation, there were some trends that are supported by the findings of other researchers. For example, by the end of the experiment, shoot matter was higher overall in the susceptible biotype, but both appeared to take advantage of the increased concentration of CO_2_^23,35–38,40,41^. Each produced more dry matter at elevated CO_2_ than in ambient conditions, even when moisture stressed. This demonstrates there was at least a small degree of positive effect from elevated CO_2_. It is sometimes referred to as the “fertilizer” effect, since plant size is increased, and some researchers^42^ have attempted to link this outcome to the possibility of increased crop yields. However, it is not yet properly understood if this is actually the advantage that it appears to be. Although plants may become larger with the increased availability of CO_2_, their biochemical makeup can be altered as well, and this may be to the detriment of crop quality. For example, it is known that nitrogen fixation is an important process for protein synthesis, but levels of protein are reduced in plants as a result of exposure to increased levels of CO_2_^36,37^. In pasture species, this may have flow-on effects, for example, to livestock. If the reduction in proteins were to become significant in pasture grasses, ensuring that adequate supplemental feeding is available from alternate protein sources would then be necessary. For *Chloris truncata*, as a grass commonly grazed by native animals, the effect of a significant reduction in protein levels would necessitate increased grazing effort. This may lead to overgrazing of native grasslands, with potentially dire consequences for native fauna populations.

The trend for inflorescence dry matter production by each biotype contrasted with those for shoot dry matter. Whilst the overall biomass production for the susceptible biotype was higher than for the resistant plants and tended to increase with increasing CO_2_ in both biotypes, the overall trend for inflorescence dry matter was somewhat mixed. The resistant population tended to produce more inflorescence dry matter with increasing CO_2_, whereas the susceptible biotype tended to produce less. However, these results were not predictive for the number of seeds produced per plant.

Given these results, it is perhaps surprising that the susceptible plants produced more seeds overall than the resistant type. In addition, in contrast to the results for inflorescence biomass, there was a consistently positive response for the number of seeds produced per plant at elevated CO_2_ compared to ambient in both biotypes. However, there was no statistical significance for this result. The overall result is higher fecundity of the susceptible biotype than in the resistant biotype.

### Trial 3: Effects of moisture stress and carbon dioxide on plant height, number of leaves and inflorescences

Although there was no statistical difference between biotypes, the trends for plant growth were similar to those for shoot dry matter and seed production. There was some initial indication of more rapid early growth in the resistant plants compared to the susceptible biotype, but this was temporary. Plant height of the former group was slightly greater than the latter at Weeks 0 and 2. However, at Week 5, the susceptible plants had largely caught up to the resistant biotype and by Week 7, the susceptible plants were taller than the resistant biotype.

The number of leaves varied according to biotype and there was a statistically significant difference between each. The susceptible plants had produced the largest number of leaves by Week 7, which was significantly more than the resistant biotype. Water treatment was a significant factor influencing this result.

For the development of inflorescences, each biotype showed variable responses to elevated CO_2_ and water stress for the initiation of inflorescence production, as well as contrasting trends in final numbers of inflorescences. The first inflorescence was produced by the resistant plants in Week 2 at elevated CO_2_ and both water treatments. By Week 7, moisture-stressed plants had produced varying numbers of inflorescences according to biotype and in response to the different levels of CO_2_. In the resistant biotype, larger numbers of inflorescences were produced in both water treatments in the elevated CO_2_ treatment than in the ambient condition. In the susceptible plants, this trend was reversed, with fewer inflorescences being produced at elevated CO_2_ compared to ambient levels in both water treatments.

Taken together, these results indicate that, like other C_4_ grasses, *C. truncata* is able to utilize increased levels of atmospheric CO_2_ to increase overall growth^21,23,40,41^, and that the susceptible biotype has a more positive response for exposure to CO_2_ than the resistant plants, in a similar manner to *Eleusine indica*^26^. Herbicide resistance, although not uniformly occurring in all populations of this species or its relatives, is of concern for future management of this plant where it occurs as a weed. The reliance on herbicides for control of *C. truncata* is likely to continue in these regions, since no-till or minimum-till approaches to cropping are becoming popular among grain producers, due to its many benefits, including, but not limited to, avoidance of soil compaction and reduction of soil erosion^43^. Therefore, it is recommended that future research be undertaken either to find alternative herbicides for control of this species or to find alternative non-chemical methods to achieve weed management.

In consideration of the results of the trial where herbicide was not used, and even though little statistical significance was found, plant dry matter production and fecundity of the resistant biotype lagged slightly behind that of the susceptible varieties, even where there was additional CO_2_ available to stimulate growth. This may perhaps suggest that there is a metabolic cost for development of resistance to herbicides^26^ in this species which may affect the outcome of competition between these two biotypes, particularly if both are growing in a situation where there is no herbicide exposure over a period of time. The higher fecundity of the susceptible biotype may thus enable it to outcompete the resistant variety in equivalent environments.

However, when considering the situation of *C. truncata* occurring in grasslands within its native range, the increase in the number of seeds and overall physical size in response to elevated CO_2_ may perhaps occasional lead to reduced concern for this species’ future, since it appears that its fecundity is not necessarily negatively impacted. However, it is clear that increasing CO_2_ levels will not occur in isolation from increased temperatures or changes in rainfall patterns. Thus, although it is considered that *C. truncata* is relatively drought resistant, future climate change outcomes may align with poorer outcomes for this species, since germination is negatively impacted by reduced moisture availability. To scope out this possibility, a trial that includes temperature variation to test for this species’ response to this factor in addition to those tested here is recommended.

## Materials and Methods

The experiment described in this article was conducted in the summer season of 2017–18 at the University of Queensland, Gatton, Australia, using two biotypes of *Chloris truncata* seeds labelled as ‘glyphosate-resistant’ and ‘glyphosate-susceptible’. Seeds were collected from agricultural fallow fields during April 2016 from Cecil Plains, transported to Gatton, and stored at room temperature until trials were commenced. To test the herbicide-resistant status of the seeds, plants were grown in a screen house during the summer of 2016–17 and sprayed with glyphosate at 720 g a.e. ha^−1^. The plants of one biotype survived herbicide application (hereafter called ‘resistant biotype’), whereas the other biotype plants were killed (hereafter called ‘susceptible biotype’). In the following summer of 2017–18, seeds from each population were germinated and grown under three pairs of conditions: moisture stress and herbicide application; CO_2_ and herbicide application; and CO_2_ and moisture stress. Moisture stress and carbon dioxide tended to decrease the effectiveness of herbicides in both biotypes. Susceptible biotype plants tended to have higher biomass and fecundity by the conclusion of the experiment than the resistant biotype.

### Trial 1: Moisture stress and herbicide application

Seeds from each of the herbicide susceptible and resistant biotypes were planted in 14-cm diameter pots, in a factorial randomised design, on December 22, 2017. The growth media was a 3:1 mixture of field soil and potting mix, with one kilogram (1 kg) of the mixture per pot. The treatments were, resistant or susceptible biotype; well-watered (WW) or water-stressed (WS) treatment; and varying herbicide concentrations, 0 (control), 180, 360, 720, 1440 g a.e. ha^−1^ of glyphosate (g a.e. = grams of acid equivalent). There were eight replications of each treatment. Immediately after emergence, the germinants were thinned to one plant per pot. The moisture stress treatment commenced on January 3, 2018. Forty of the plants in each population continued to be watered daily with a sprinkler system, but the water was withheld from the remainder for 12 days (until January 15), at which time the glyphosate treatment was applied. Observations of plant percentage survival were made on January 29, two weeks after herbicide application. Plants were harvested and measurement of shoot dry matter subsequently recorded.

### Trial 2: Carbon dioxide and herbicide application

Seeds from each biotype were planted in 5 cm diameter pots, in a 3:1 mixture of field soil and potting mix, on December 22, 2017. Smaller pots were used in this study as all pots needed to fit inside the growth chamber used. Forty replicates from each population were placed into environmental growth chambers (Climatron®, Thermoline Scientific), which were set to either ambient (450 ppm) or elevated (750 ppm) levels of CO_2_. Pots were sub-irrigated in this trial.

Although the “ambient” CO_2_ level chosen for this experiment was higher than the current atmospheric concentration of 410 ppm as measured in June 2018 at the Mauna Loa Observatory in Hawaii^44^ a few months after the trials were conducted (between December 2017 to March 2018), 450 ppm was the minimum possible concentration setting for CO_2_ in the equipment used for this experiment. This CO_2_ concentration provides a valid comparison, as a baseline, to contrast with the potential effects on this species of future, elevated, atmospheric CO_2_ concentrations.

On January 20, 2018, when seedlings had reached the four-leaf stage, they were sprayed with glyphosate at five different concentrations, 0 (control), 180, 360, 720, and 1440 g a.e. ha^−1^. Observations of the effects of both treatments on the percentage of plant survival were made on February 10, 2018, three weeks after herbicide application. Plants were harvested and shoot dry matter recorded.

### Trial 3: Carbon dioxide and moisture stress

Twenty-four replicates of seeds from each of the herbicide susceptible and herbicide resistant populations were planted on December 22, 2017. The seeds were planted in 14 cm diameter pots, in a 3:1 mixture of field soil and potting mix, with one kilogram (1 kg) of the mixture per pot. For each population, twelve pots were placed into environmental growth chambers set to either ambient (450 ppm) or elevated (750 ppm) CO_2_. Plants were grown in well-watered conditions until January 12. After this date, six plants of each population were watered to 100% field capacity and the remainder watered to 50% field capacity, until the conclusion of the experiment. Water was supplied to the plants in each growth chamber at a 3-d interval after weighing the pots. Prior to re-watering, the soil moisture range was 10–15% in the 50% treatment and 38–40% in the 100% treatment. Commencing when water was reduced for half of the plants, observations of plant height, number of leaves, and inflorescences were recorded in weeks 0, 2, 5 and 7 (January 12 and 26, February 16, and March 2). The plants were harvested on March 2 to measure shoot and inflorescence dry matter mass, and the number of seeds produced per plant was also recorded at this time.

### Data analyses

Data were analysed according to the parameters tested for each treatment regime. A three-way ANOVA was done with SuperAnova for all the data collected in the first two trials, as well as shoot dry matter, inflorescence dry matter and numbers of seeds produced per plant in the third trial. A repeated measures ANOVA was done with SPSS (Version 25) for plant height, numbers of leaves and inflorescences in the third trial.

## Supplementary information

Dataset 1
