# Supplementary material for: Response of Chloris truncata to moisture stress, elevated carbon dioxide and herbicide application
Source: Sci Rep. 2019 Jul 24;9:10721. doi: 10.1038/s41598-019-47237-x (PMC6656741; doi:10.1038/s41598-019-47237-x)
Supplement: Supplementary file 1 — Dataset 1 [file 41598_2019_47237_MOESM1_ESM.docx]

**Response of *Chloris truncata* to moisture stress, elevated carbon dioxide and herbicide application**

S. L. Weller^1^, S. K. Florentine^1^, N. K. Mutti^2^, Prashant Jha^3^, Bhagirath S. Chauhan^2*^

^1^Centre for Environmental Management, School of Health and Life Sciences, Federation University Australia, Mt Helen, Ballarat, PO Box 663, Vic 3350, Australia

^2^Centre for Crop Science, Queensland Alliance for Agriculture and Food Innovation (QAAFI), The University of Queensland, Gatton, Queensland 4343, Australia

^3^Southern Agricultural Research Centre, Montana State University, Huntley, MT 59037, United States of America

**Correspondence to:* b.chauhan@uq.edu.au

**SUPPLEMENTARY DATA**

**Trial 1 - Water Stress and Herbicide Application**

**Table S1**: Plant survival according to biotype, water treatment and herbicide application rate.

|  | Water stress + Herbicide | | | |
| --- | --- | --- | --- | --- |
| Herbicide | R + WW | R + WS | S + WW | S + WS |
| 0 g.a.i./ha | 100% | 100% | 100% | 100% |
| 180 g.a.i./ha | 100% | 100% | 100% | 100% |
| 360 g.a.i./ha | 100% | 100% | 0% | 100% |
| 720 g.a.i./ha | 100% | 100% | 0% | 100% |
| 1440 g.a.i./ha | 100% | 100% | 0% | 100% |

**Trial 2 - Carbon Dioxide and Herbicide Application**

**Table S2**: Plant survival according to biotype, carbon dioxide and herbicide application rate.

|  | Carbon dioxide + Herbicide | | | |
| --- | --- | --- | --- | --- |
| Herbicide | R + 450 ppm | R + 750 ppm | S + 450 ppm | S + 750 ppm |
| 0 g.a.i./ha | 100% | 100% | 100% | 100% |
| 180 g.a.i./ha | 100% | 100% | 100% | 100% |
| 360 g.a.i./ha | 100% | 100% | 0% | 25% |
| 720 g.a.i./ha | 100% | 100% | 0% | 0% |
| 1440 g.a.i./ha | 25% | 62.5% | 0% | 0% |

**Trial 3 – Water Stress and Carbon Dioxide**

**Table S3**: Plant height according to biotype, water treatment, carbon dioxide and time (weeks).

| Population + water treatment + CO_2_ treatment + Week | Mean + SE |
| --- | --- |
| Resistant + 50% + 450 ppm + Week 0 | 14.17 + 0.77 |
| Resistant + 50% + 450 ppm + Week 2 | 37.28 + 1.28 |
| Resistant + 50% + 450 ppm + Week 5 | 44.00 + 1.45 |
| Resistant + 50% + 450 ppm + Week 7 | 58.17 + 2.12 |
| Resistant + 50% + 750 ppm + Week 0 | 17.83 + 2.06 |
| Resistant + 50% + 750 ppm + Week 2 | 39.02 + 1.48 |
| Resistant + 50% + 750 ppm + Week 5 | 48.67 + 0.84 |
| Resistant + 50% + 750 ppm + Week 7 | 64.17 + 3.47 |
| Resistant + 100% + 450 ppm + Week 0 | 14.33 + 1.29 |
| Resistant + 100% + 450 ppm + Week 2 | 36.72 + 1.58 |
| Resistant + 100% + 450 ppm + Week 5 | 48.17 + 1.93 |
| Resistant + 100% + 450 ppm+ Week 7 | 57.67 + 1.24 |
| Resistant + 100% + 750 ppm + Week 0 | 16.50 + 1.70 |
| Resistant + 100% + 750 ppm + Week 2 | 41.25 + 1.96 |
| Resistant + 100% + 750 ppm + Week 5 | 47.50 + 2.18 |
| Resistant + 100% + 750 ppm+ Week 7 | 68.42 + 3.51 |
| Susceptible + 50% + 450 ppm + Week 0 | 11.58 + 0.43 |
| Susceptible + 50% + 450 ppm + Week 2 | 33.57 + 0.97 |
| Susceptible + 50% + 450 ppm + Week 5 | 50.33 + 2.09 |
| Susceptible + 50% + 450 ppm+ Week 7 | 66.42 + 3.17 |
| Susceptible + 50% + 750 ppm + Week 0 | 11.08 + 0.69 |
| Susceptible + 50% + 750 ppm + Week 2 | 31.83 + 1.48 |
| Susceptible + 50% + 750 ppm + Week 5 | 48.33 + 2.57 |
| Susceptible + 50% + 750 ppm+ Week 7 | 73.00 + 5.37 |
| Susceptible + 100% + 450 ppm + Week 0 | 11.58 + 0.88 |
| Susceptible + 100% + 450 ppm + Week 2 | 34.27 + 1.92 |
| Susceptible + 100% + 450 ppm + Week 5 | 48.83 + 2.60 |
| Susceptible + 100% + 450 ppm+ Week 7 | 74.08 + 2.33 |
| Susceptible + 100% + 750 ppm + Week 0 | 10.00 + 0.58 |
| Susceptible + 100% + 750 ppm + Week 2 | 30.75 + 2.98 |
| Susceptible + 100% + 750 ppm + Week 5 | 48.67 + 4.51 |
| Susceptible + 100% + 750 ppm+ Week 7 | 74.50 + 2.18 |

**Table S4**: Number of leaves according to biotype, water treatment, carbon dioxide and time (weeks).

| Population + water treatment + CO_2_ treatment + Week | Mean + SE |
| --- | --- |
| Resistant + 50% + 450 ppm + Week 0 | 12.17 + 1.14 |
| Resistant + 50% + 450 ppm + Week 2 | 32.83 + 3.90 |
| Resistant + 50% + 450 ppm + Week 5 | 75.17 + 10.42 |
| Resistant + 50% + 450 ppm + Week 7 | 83.67 + 5.81 |
| Resistant + 50% + 750 ppm + Week 0 | 17.00 + 2.07 |
| Resistant + 50% + 750 ppm + Week 2 | 36.00 + 5.23 |
| Resistant + 50% + 750 ppm + Week 5 | 94.00 + 11.55 |
| Resistant + 50% + 750 ppm + Week 7 | 93.50 + 4.81 |
| Resistant + 100% + 450 ppm + Week 0 | 12.00 + 0.88 |
| Resistant + 100% + 450 ppm + Week 2 | 35.83 + 3.23 |
| Resistant + 100% + 450 ppm + Week 5 | 84.50 + 14.32 |
| Resistant + 100% + 450 ppm+ Week 7 | 117.67 + 10.45 |
| Resistant + 100% + 750 ppm + Week 0 | 14.17 + 1.77 |
| Resistant + 100% + 750 ppm + Week 2 | 47.67 + 4.14 |
| Resistant + 100% + 750 ppm + Week 5 | 115.00 + 11.62 |
| Resistant + 100% + 750 ppm+ Week 7 | 132.17 + 17.71 |
| Susceptible + 50% + 450 ppm + Week 0 | 13.67 + 0.84 |
| Susceptible + 50% + 450 ppm + Week 2 | 36.50 + 1.61 |
| Susceptible + 50% + 450 ppm + Week 5 | 100.33 + 8.84 |
| Susceptible + 50% + 450 ppm+ Week 7 | 136.50 + 7.68 |
| Susceptible + 50% + 750 ppm + Week 0 | 11.83 + 1.42 |
| Susceptible + 50% + 750 ppm + Week 2 | 34.17 + 3.92 |
| Susceptible + 50% + 750 ppm + Week 5 | 121.33 + 9.99 |
| Susceptible + 50% + 750 ppm+ Week 7 | 140.67 + 9.56 |
| Susceptible + 100% + 450 ppm + Week 0 | 13.67 + 1.19 |
| Susceptible + 100% + 450 ppm + Week 2 | 43.50 + 4.34 |
| Susceptible + 100% + 450 ppm + Week 5 | 125.17 + 20.71 |
| Susceptible + 100% + 450 ppm+ Week 7 | 164.50 + 14.22 |
| Susceptible + 100% + 750 ppm + Week 0 | 11.50 + 0.81 |
| Susceptible + 100% + 750 ppm + Week 2 | 31.33 + 4.34 |
| Susceptible + 100% + 750 ppm + Week 5 | 106.33 + 15.72 |
| Susceptible + 100% + 750 ppm+ Week 7 | 167.00 + 17.19 |

**Table S5**: Number of inflorescences according to biotype, water treatment, carbon dioxide and time (weeks).

| Population + water treatment + CO_2_ treatment + Week | Mean + SE |
| --- | --- |
| Resistant + 50% + 450 ppm + Week 0 | 0 + 0 |
| Resistant + 50% + 450 ppm + Week 2 | 0 + 0 |
| Resistant + 50% + 450 ppm + Week 5 | 1.67 + 0.56 |
| Resistant + 50% + 450 ppm + Week 7 | 5.00 + 0.78 |
| Resistant + 50% + 750 ppm + Week 0 | 0 + 0 |
| Resistant + 50% + 750 ppm + Week 2 | 0.33 + 0.19 |
| Resistant + 50% + 750 ppm + Week 5 | 2.67 + 0.73 |
| Resistant + 50% + 750 ppm + Week 7 | 7.00 + 0.47 |
| Resistant + 100% + 450 ppm + Week 0 | 0 + 0 |
| Resistant + 100% + 450 ppm + Week 2 | 0 + 0 |
| Resistant + 100% + 450 ppm + Week 5 | 1.17 + 0.28 |
| Resistant + 100% + 450 ppm+ Week 7 | 5.17 + 0.80 |
| Resistant + 100% + 750 ppm + Week 0 | 0 + 0 |
| Resistant + 100% + 750 ppm + Week 2 | 0.33 + 0.19 |
| Resistant + 100% + 750 ppm + Week 5 | 1.17 + 0.44 |
| Resistant + 100% + 750 ppm+ Week 7 | 5.83 + 0.95 |
| Susceptible + 50% + 450 ppm + Week 0 | 0 + 0 |
| Susceptible + 50% + 450 ppm + Week 2 | 0 + 0 |
| Susceptible + 50% + 450 ppm + Week 5 | 1.33 + 0.19 |
| Susceptible + 50% + 450 ppm+ Week 7 | 6.50 + 0.74 |
| Susceptible + 50% + 750 ppm + Week 0 | 0 + 0 |
| Susceptible + 50% + 750 ppm + Week 2 | 0 + 0 |
| Susceptible + 50% + 750 ppm + Week 5 | 1.33 + 0.19 |
| Susceptible + 50% + 750 ppm+ Week 7 | 6.17 + 1.14 |
| Susceptible + 100% + 450 ppm + Week 0 | 0 + 0 |
| Susceptible + 100% + 450 ppm + Week 2 | 0 + 0 |
| Susceptible + 100% + 450 ppm + Week 5 | 1.50 + 0.31 |
| Susceptible + 100% + 450 ppm+ Week 7 | 7.50 + 1.85 |
| Susceptible + 100% + 750 ppm + Week 0 | 0 + 0 |
| Susceptible + 100% + 750 ppm + Week 2 | 0 + 0 |
| Susceptible + 100% + 750 ppm + Week 5 | 0.67 + 0.30 |
| Susceptible + 100% + 750 ppm+ Week 7 | 5.67 + 1.59 |

**Table S6:** Inflorescence dry matter according to biotype, water treatment and carbon dioxide.

| Population + water treatment + CO_2_ treatment | Mean + SE |
| --- | --- |
| Resistant + 50% + 450 ppm | 0.77 + 0.12 |
| Resistant + 50% + 750 ppm | 0.90 + 0.17 |
| Resistant + 100% + 450 ppm | 0.88 + 0.16 |
| Resistant + 100% + 750 ppm | 1.29 + 0.22 |
| Susceptible + 50% + 450 ppm | 1.06 + 0.08 |
| Susceptible + 50% + 750 ppm | 0.84 + 0.18 |
| Susceptible + 100% + 450 ppm | 1.45 + 0.30 |
| Susceptible + 100% + 750 ppm | 1.01 + 0.34 |
